# Chitosan Oligosaccharides Alleviate Heat-Stress-Induced Lipid Metabolism Disorders by Suppressing the Oxidative Stress and Inflammatory Response in the Liver of Broilers

**DOI:** 10.3390/antiox12081497

**Published:** 2023-07-27

**Authors:** Ruixia Lan, Huiwen Luo, Fan Wu, Yuchen Wang, Zhihui Zhao

**Affiliations:** Department of Animal Science and Technology, College of Coastal Agriculture Sciences, Guangdong Ocean University, Zhanjiang 524088, China; lanrx@gdou.edu.cn (R.L.); luohuiwen@stu.gdou.edu.cn (H.L.); wufan21@stu.gdou.edu.cn (F.W.); wangyuchen@stu.gdou.edu.cn (Y.W.)

**Keywords:** chitosan oligosaccharides, liver, lipid metabolism, oxidative stress, inflammation reaction, heat-stressed broilers

## Abstract

Heat stress has been reported to induce hepatic oxidative stress and alter lipid metabolism and fat deposition in broilers. Chitosan oligosaccharides (COSs), a natural oligosaccharide, has anti-oxidant, anti-inflammatory, and lipid-lowering effects. This study is conducted to evaluate dietary COS supplementation on hepatic anti-oxidant capacity, inflammatory response, and lipid metabolism in heat-stressed broilers. The results indicate that heat-stress-induced poor (*p* < 0.05) growth performance and higher (*p* < 0.05) abdominal adiposity are alleviated by COS supplementation. Heat stress increases (*p* < 0.05) serum AST and ATL activity, serum and liver MDA, TG, TC, and LDL-C levels, and the expression of hepatic *IL-1β*, *IL-6*, *SREBP-1c*, *ACC*, and *FAS*, while it decreases (*p* < 0.05) serum SOD and CAT activity, liver GSH-Px and SOD activity, and the expression of hepatic *Nrf2*, *GPX1*, *IL-10*, *MTTP*, *PPARα*, and *CPT1.* Nevertheless, COS supplementation decreases (*p* < 0.05) serum AST and ATL activity, serum and liver MDA, TG, TC, and LDL-C levels, and the expression of hepatic *IL-1β*, *IL-6*, *SREBP-1c*, *ACC*, and *FAS*, while it increases (*p* < 0.05) serum SOD and CAT activity, liver GSH-Px activity, and the expression of hepatic *Nrf2*, *CAT*, *IL-10*, *LPL*, *MTTP*, *PPARα*, and *CPT1.* In conclusion, COS could alleviate heat-stress-induced lipid metabolism disorders by enhancing hepatic anti-oxidant and anti-inflammatory capacity.

## 1. Introduction

Decades of intensive genetic selection have led to improvements in broiler growth rate and meat production; however, these have been accompanied by high fat deposition, metabolic disorders, and heat stress sensitivity [1,2,3]. With the increasing global temperature, heat stress has become more frequent, inducing numerous economic losses in the poultry industry by suppressing growth performance, influencing metabolism, health status, meat quality, and mortality [4,5,6,7]. Broilers, especially fast-growing meat broilers, are sensitive to heat stress due to their high metabolic rate, fast growth rate, being feather-covered, and lack of sweat glands [8]. The liver is one of the most important metabolic organs, having an irreplaceable role in digestion, metabolism, immunity, and detoxification [9]. The liver is sensitive and responsive to heat stress, thus influencing hepatic lipid metabolism [4,5,9,10,11]. Heat-stress-induced hepatic lipid metabolism disorders have received increasing attention due to their impacts on production in modern commercial broilers [7]. In poultry, over 95% of fatty acid de novo synthesis is completed in the liver, and the adipose tissue serves as a lipid storage site. The fat deposition depends on triacylglycerol (TG) synthesis in the liver, the TG-rich lipoproteins that transports TG from the liver to extrahepatic tissues, and serum TG utilization. Previous literature has indicated that heat-stress-induced fat deposition occurs by promoting hepatic lipogenesis and inhibiting lipolysis in broilers [4,5]. Thus, the excessive abdominal adiposity of heat-stressed broilers may be associated with hepatic lipid synthesis, TG transports, and serum TG utilization [7,12]. Previous studies have also indicated that a beneficial way to decrease fat deposition is to decrease fatty acid synthesis, and promote fatty acid oxidation [13]. Sterol regulatory element-binding protein 1c (*SREBP-1c*) has a key role in regulating the expression of fatty-acid-synthesis-related gene, including acetyl-coenzyme carboxylase (*ACC*), fatty acid synthase (*FAS*), elongation of very long chain fatty acids 6 (*ELOVA6*), and stearoyl-CoA desaturase (*SCD*) [14]. Meanwhile, peroxisome proliferator-activated regulator α (*PPARα*) has a key role in hepatic lipid metabolism as it regulates the expression of lipolysis, transportation, and fatty-acid-oxidation-related gene, such as carnitine palmitoyltransferase-1 (*CPT1*), microsomal triglyceride transfer protein (*MTTP*), lipoprotein lipase (*LPL*), and fatty acid binding protein-1 (*FABP1*) [15]. Furthermore, heat stress also induces liver damage by down-regulating the expression of the anti-oxidative-related gene, such as nuclear factor erythroid related factor 2 (*Nrf2*), heme oxygenase-1 (*HO-1*), superoxide dismutase (*SOD*), or glutathione peroxidase (*GPX1*), as well as up-regulating the pro-inflammatory-related gene, such as tumor necrosis factor-*α* (*TNF-α*) and interleukin-6 (*IL-6*) in broilers [10,11,16,17,18]. These findings demonstrate that heat stress affects hepatic-lipid-metabolism-related gene expression, which is associated with changes in the expression of oxidative stress and inflammatory-response-related gene. Nutritional management, especially feed additives, which exerts lipid-lowering, anti-oxidant, and/or anti-inflammatory effects, might represent a beneficial regulator to alleviate heat-stress-induced hepatic lipid metabolism disorders. For instance, itaconate and astaxanthin, which exert an anti-oxidant and anti-inflammatory capacity, were used to alleviate heat-stress-induced hepatic lipid metabolism disorders in broilers [18,19].

Chitosan oligosaccharides (COSs), a degradation product of chitin or chitosan via enzyme or acid hydrolysis, are characterized by an average molecular weight of less than 3900 Da, a degree of polymerization less than 20%, and a degree of deacetylation over 90% [20]. Recently, COS has received widespread attention due to its excellent anti-oxidant and anti-inflammatory capacity, lipid-lowering effects by decreasing serum lipid levels, hepatic lipid accumulation, fat deposition, and regulation of lipogenesis- and lipolysis-related gene expression in cell or animal models [20,21,22,23,24,25]. Additionally, Choi et al. [26] reported that COS decreased serum and hepatic lipid profiles in mice fed a high-fat diet, and Bai et al. [27] indicated that COS ameliorated hepatic glucolipid metabolism disorders by alleviating the inflammatory response in mice fed a high-fat diet. Tao et al. [28] demonstrated that COS regulated hepatic lipid metabolism and alleviated the hepatic inflammatory response and oxidative stress in nonalcoholic fatty liver disease mice. Furthermore, our previous studies also demonstrated that COS could ameliorate the inflammatory response and oxidative stress induced by heat stress (34 °C, 8 h/day) on the liver, intestinal, and breast muscle of yellow-feather broilers [29,30,31]. However, there are limited studies focusing on the effects of dietary COS supplementation on hepatic lipid metabolism in heat-stressed broilers and the underlying mechanism. It is unknown whether the lipid-lowering effects is associated with the anti-oxidant and anti-inflammatory capacity of COS. Therefore, the purpose of this study is to evaluate the effects of dietary COS supplementation on the hepatic anti-oxidant capacity, inflammatory response, and lipid metabolism in heat-stressed broilers.

## 2. Materials and Methods

### 2.1. Animals and Experiment Design

Totally, 250 1-day-old male Arbor Acres broilers were purchased from the local commercial hatchery (Suixi, China) and given commercial standard diets and management from day 1 to 14. On day 14, 180 broilers were randomly selected and allocated to 3 groups with 6 replication pens and 10 broilers per pen for this 28-day experiment. The groups were: CON group, with a basal diet, and raised in temperature-controlled room (24 ± 1 °C); HS and HSC_600_ groups were raised in high-temperature-controlled room (34 ± 1 °C, 8 h/day and 24 ± 1 °C, 16 h/day) and fed the basal diet with 0 or 600 mg/kg COS (dose based on our preliminary experiment). The basal diet was formulated to meet or exceed the recommendation of the National Research Council (Table S1). The feed was provided in mash form, with COS supplementation at the expense of corn. COS was purchased from Qingdao Songtian Biotechnology Co., Ltd., Qingdao, China (purity ≥ 90%, deacetylation degree ≥ 90%, and average molecular weight ≤ 3000 Da).

### 2.2. Growth Performance

On the beginning (day 1, aged 14-day) and the end day (day 28, aged 42-day) of the experiment, feed intake and body weight (BW) were recorded as pen basis to calculate average daily feed intake (ADFI), average daily gain (ADG), and feed conversion ratio (FCR).

### 2.3. Sampling

On the end day of the experiment, after 8 h fasting, one broiler from each replication pen was randomly selected (6 broilers/treatment). After weight individually, blood samples were collected from the brachial vein and centrifuged at 3500× *g* for 15 min at 4 °C; the serum samples were collected and stored at −20 °C until analysis. After that, broilers were exterminated by cervical dislocation and exsanguinated, about 1 g (2 copies) liver sample was collected, snap-frozen in liquid nitrogen quickly, and stored at −80 °C for later analysis. Finally, liver and abdominal adipose weight (including the cloaca- and gizzard-surrounded fat) was recorded, and the relative weight of liver and abdominal adipose was calculated as the percentage of BW.

### 2.4. Aminotransferase, Anti-Oxidant, and Lipid Metabolism Parameters

Serum alanine aminotransferase (ALT, NO. C009-2-1), aspartate aminotransferase (AST, NO. C010-2-1), malondialdehyde (MDA, NO. A003-4-1), glutathione peroxidase (GSH-Px, NO. A005-1-2), superoxide dismutase (SOD, NO. A001-1-2), catalase (CAT, NO. A007-2-1), TG (NO. BC0625), total cholesterol (TC, NO. A111-1-1), low density lipoprotein cholesterol (LDL-C, NO. A113-1-1), and high density lipoprotein cholesterol (HDL-C, NO. A112-1-1) were detected using commercial kits (Nanjing Jiancheng Bioengineering Institute, Nanjing, China) according to the instructions.

For liver parameter analyzing, about 0.5 g sample was homogenized in 4.5 mL ice-cold phosphate-buffered saline (PBS) and centrifuged at 3500× *g* for 15 min at 4 °C to collect the 10% liver supernatant to analyze the content of MDA, SOD, GSH-Px, CAT, TG, TC, HDL-C, and LDL-C, and the protein content was determined using the total protein assay kit (NO. A045-4-2, Nanjing Jiancheng Bioengineering Institute, Nanjing, China). The protein concentration of 10% liver supernatant was used to standardize these parameters.

### 2.5. Real-Time Quantitative PCR

Total mRNA extraction, cDNA reverse transcription, and quantitative real-time polymerase chain reaction (RT-PCR) analysis were performed, following the methods described in our previous study [32]. The primers used were described in former literature [4,31,33] and listed in Table S2. The PCR reactions were performed in triplicate, and the results were normalized to the β-actin expression and calculated by 2^−∆∆Ct^ method [34].

### 2.6. Statistical Analysis

The pen was used as the experiment unit and the data were presented as mean ± standard error. SAS 2003 (v. 9.1, SAS Institute Inc., Cary, NC, USA) was used to perform one-way analysis of variance (ANOVA) followed by Duncan’s multiple range test. Spearman’s correlation was analyzed using SPSS software (v. 20.0, SPSS Inc., Chicago, IL, USA). *p* < 0.05 was considered as significant difference between the values.

## 3. Results

### 3.1. Growth Performance, and Liver and Abdominal Adipose Index

Compared to the CON group, heat stress induced a lower (*p* < 0.05) final BW, ADG, and ADFI, and higher (*p* < 0.05) FCR, and absolute and relative weight of abdominal adipose (Table 1). Compared to the HS group, COS supplementation increased the final BW, ADG, ADFI, whereas it lowered the (*p* < 0.05) FCR, and absolute and relative weight of abdominal adipose. No significant differences were observed in the initial BW, and absolute or relative weight of the liver among the groups.

### 3.2. Liver Function Index

**Figure 1:**
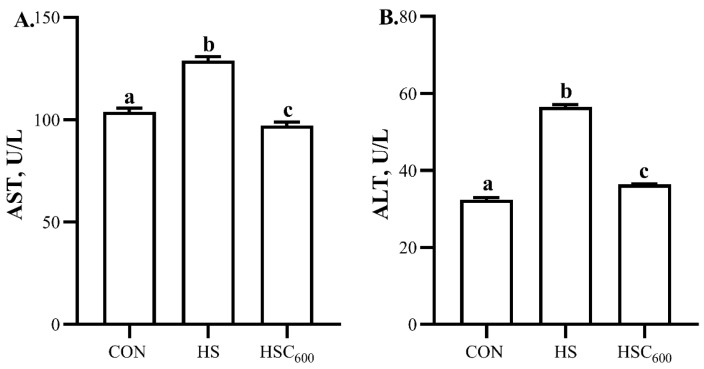
Dietary chitosan oligosaccharide supplementation on liver damage index in broilers under heat stress. (**A**), AST level; (**B**), ALT level; AST, aspartate aminotransferase; ALT, alanine aminotransferase; CON, basal diet and raised in temperature-controlled room (24 ± 1 °C); HS and HSC_600_ were raised in high-temperature-controlled room (34 ± 1 °C, 8 h/day and 24 ± 1 °C, 16 h/day) and fed the basal diet with 0 or 600 mg/kg chitosan oligosaccharides; ^a, b, c^ Means with different superscripts indicated significantly different (*p* < 0.05).

Compared to the CON group, heat stress induced higher (*p* < 0.05) serum ALT and AST activity (Figure 1). COS supplementation attenuated the heat-stress-induced higher (*p* < 0.05) serum ALT and AST activity.

### 3.3. Anti-Oxidant Capacity

**Figure 2:**
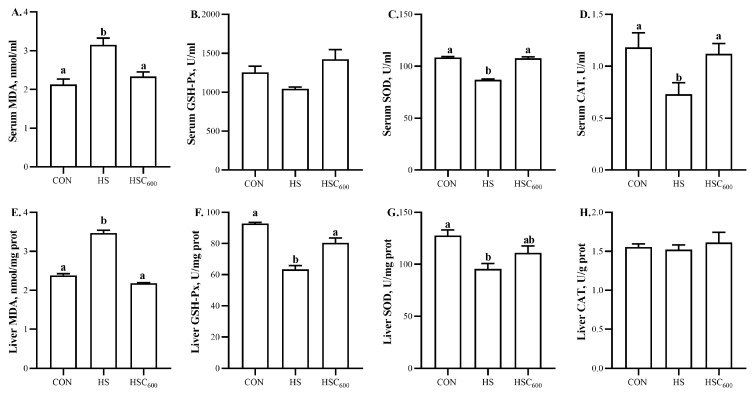
Dietary chitosan oligosaccharide supplementation on anti-oxidant capacity of serum and liver in broilers under heat stress. (**A**), Serum MDA level; (**B**), Serum GSH-Px activity; (**C**), Serum SOD activity; (**D**), Serum CAT activity; (**E**), Hepatic MDA level; (**F**), Hepatic GSH-Px activity; (**G**), Hepatic SOD activity; (**H**), Hepatic CAT activity; MDA, malondialdehyde; GSH-Px, glutathione peroxidase; SOD, superoxide dismutase; CAT, catalase; CON, basal diet and raised in temperature-controlled room (24 ± 1 °C); HS and HSC_600_ were raised in high-temperature-controlled room (34 ± 1 °C, 8 h/day and 24 ± 1 °C, 16 h/day) and fed the basal diet with 0 or 600 mg/kg chitosan oligosaccharides; ^a, b^ Means with different superscripts indicated significantly different (*p* < 0.05).

Compared to the CON group, heat stress induced higher (*p* < 0.05) serum and liver MDA levels, while it induced lower (*p* < 0.05) serum SOD and CAT activity, and lower liver GSH-Px and SOD activity (Figure 2). Compared to the HS group, COS supplementation induced lower (*p* < 0.05) serum and liver MDA levels, while it induced higher (*p* < 0.05) serum SOD and CAT activity, and higher liver GSH-Px activity.

**Figure 3:**
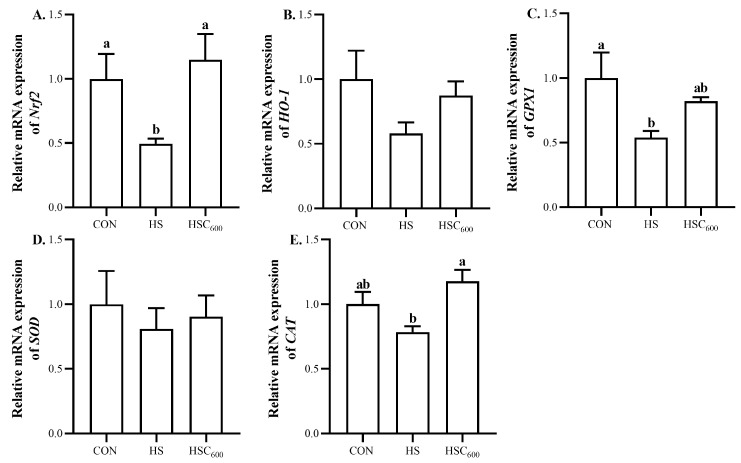
Dietary chitosan oligosaccharide supplementation on anti-oxidant-related gene expression in liver in broilers under heat stress. (**A**), *Nrf2* expression level; (**B**), *HO-1* expression level; (**C**), *GPX1* expression level; (**D**), *SOD* expression level; (**E**), *CAT* expression level; *Nrf2*, nuclear factor erythroid related factor 2; *HO-1*, heme oxygenase-1; *GPX1*, glutathione peroxidase; *SOD*, superoxide dismutase; *CAT*, catalase; CON, basal diet and raised in temperature-controlled room (24 ± 1 °C); HS and HSC_600_ were raised in high-temperature-controlled room (34 ± 1 °C, 8 h/day and 24 ± 1 °C, 16 h/day) and fed the basal diet with 0 or 600 mg/kg chitosan oligosaccharides; ^a, b^ Means with different superscripts indicated significantly different (*p* < 0.05).

Furthermore, the oxidative-stress-related gene expression was analyzed. Compared to the CON group, HS down-regulated (*p* < 0.05) the expression of *Nrf2* and *GPX1* (Figure 3). Compared with the HS group, COS supplementation up-regulated (*p* < 0.05) the expression of *Nrf2* and *CAT*. No significant differences were observed in the expression of *SOD* among the groups.

### 3.4. Inflammatory Response

**Figure 4:**
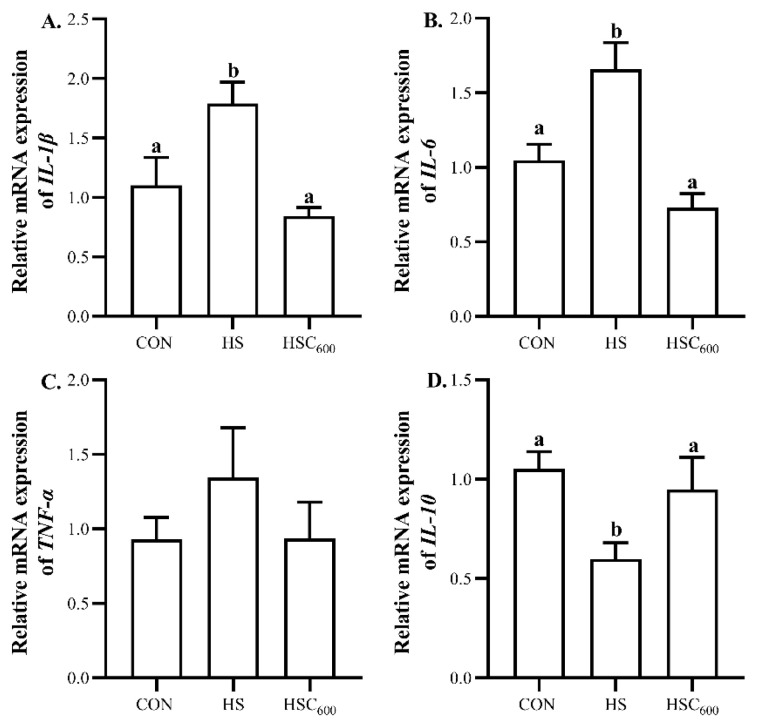
Dietary chitosan oligosaccharide supplementation on inflammation-related gene expression in liver in broilers under heat stress. (**A**), *IL-1β* expression level; (**B**), *IL-6* expression level; (**C**), *TNF-α* expression level; (**D**), *IL-10* expression level; *IL*, interleukin; *TNF-α*, tumor necrosis factor-alpha; CON, basal diet and raised in temperature-controlled room (24 ± 1 °C); HS and HSC_600_ were raised in high-temperature-controlled room (34 ± 1 °C, 8 h/day and 24 ± 1 °C, 16 h/day) and fed the basal diet with 0 or 600 mg/kg chitosan oligosaccharides; ^a, b^ Means with different superscripts indicated significantly different (*p* < 0.05).

Compared to the CON group, heat stress up-regulated (*p* < 0.05) the expression of *IL-1β* and *IL-6*, whereas it down-regulated (*p* < 0.05) the expression of *IL-10* (Figure 4). Compared to the HS group, COS supplementation decreased (*p* < 0.05) the expression of *IL-1β* and *IL-6*, whereas it increased (*p* < 0.05) the expression of *IL-10* (Figure 4). No significant differences were observed in the expression of *TNF-α* among the groups.

### 3.5. Lipid Metabolism

**Figure 5:**
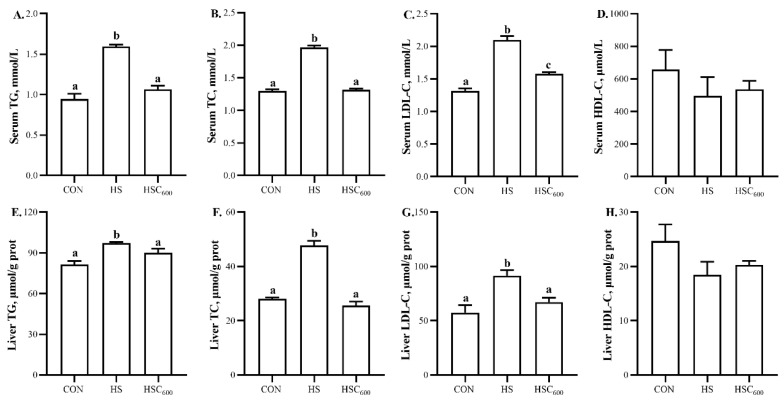
Dietary chitosan oligosaccharide supplementation on lipid metabolism parameters in broilers under heat stress. (**A**), Serum TG level; (**B**), Serum TC level; (**C**), Serum LDL-C level; (**D**), Serum HDL-C level; (**E**), Liver TG level; (**F**), Liver TC level; (**G**), Liver LDL-C level; (**H**), Liver HDL-C level; TG, triacylglycerols; TC, total cholesterol; LDL-C, low density lipoprotein cholesterol; HDL-C, high density lipoprotein cholesterol; CON, basal diet and raised in temperature-controlled room (24 ± 1 °C); HS and HSC_600_ were raised in high-temperature-controlled room (34 ± 1 °C, 8 h/day and 24 ± 1 °C, 16 h/day) and fed the basal diet with 0 or 600 mg/kg chitosan oligosaccharides; ^a, b, c^ Means with different superscripts indicated significantly different (*p* < 0.05).

Compared to the CON group, heat stress induced higher (*p* < 0.05) serum and liver TG, TC, and LDL-C levels (Figure 5). COS supplementation attenuated the heat-stress-induced higher (*p* < 0.05) serum and liver TG, TC, and LDL-C levels. No significant differences were observed in serum and liver HDL-C levels among the groups.

**Figure 6:**
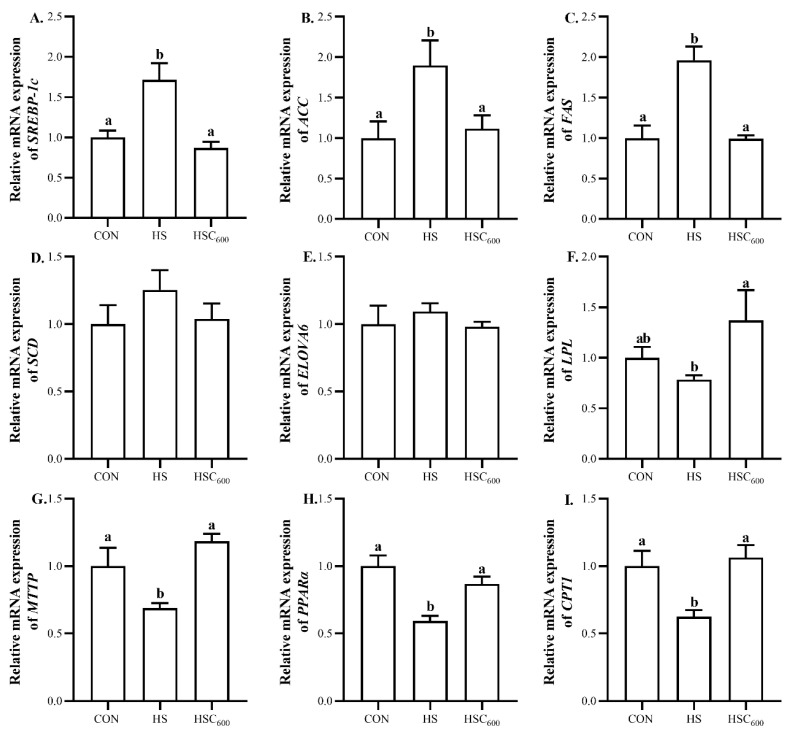
Dietary chitosan oligosaccharide supplementation on hepatic-lipid-metabolism-related gene expression in broilers under heat stress. (**A**), *SREBP-1c* expression level; (**B**), *ACC* expression level; (**C**), *FAS* expression level; (**D**), *SCD* expression level; (**E**), *ELOVA6* expression level; (**F**), *LPL* expression level; (**G**), *MTTP* expression level; (**H**), *PPARα* expression level; (**I**), *CPT1* expression level; *SREBP-1c*, sterol regulatory element-binding protein 1c; *ACC*, acetyl-coenzyme carboxylase; *FAS*, fatty acid synthase; *SCD*, stearoyl-CoA desaturase; *ELOVA6*, elongation of very long chain fatty acids 6; *LPL*, lipoprotein lipase; *MTTP*, microsomal triglyceride transfer protein; *PPARα*, proliferator-activated regulator α; *CPT1*, carnitine palmitoyltransferase-1; CON, basal diet and raised in temperature-controlled room (24 ± 1 °C); HS and HSC_600_ were raised in high-temperature-controlled room (34 ± 1 °C, 8 h/day and 24 ± 1 °C, 16 h/day) and fed the basal diet with 0 or 600 mg/kg chitosan oligosaccharides; ^a, b^ Means with different superscripts indicated significantly different (*p* < 0.05).

To further evaluate the underlying mechanisms of COS-mediated hepatic lipid metabolism, the expression of the lipid-metabolism-related gene was analyzed. Compared to the CON group, heat stress increased (*p* < 0.05) the expression of *SREBP-1c*, *ACC*, and *FAS*, whereas it decreased (*p* < 0.05) the expression of *MTTP*, *PPARα*, and *CPT1* (Figure 6). Compared to the HS group, COS supplementation decreased (*p* < 0.05) the expression of *SREBP-1c*, *ACC*, and *FAS*, whereas it increased (*p* < 0.05) the expression of *LPL*, *MTTP*, *PPARα*, and *CPT1*. No significant differences were observed in the expression of *SCD* or *ELOVA6* among the groups.

### 3.6. Correlation between Hepatic-Lipid-Metabolism-Related Gene and Indices Related to Oxidative Stress and Inflammatory Response

To further explore the correlation between hepatic-lipid-metabolism-related gene expression and the oxidative stress or the inflammatory response, Spearman’s correlation coefficients were detected (Table 2). The hepatic MDA level was positively (*p* < 0.05) correlated with the expression of *SREBP-1c*, *ACC*, and *FAS*, whereas it was negatively (*p* < 0.05) correlated with the expression of *LPL*, *MTTP*, *CPT1*, and *PPARα*. The hepatic GSH-Px level was negatively (*p* < 0.05) correlated with the expression of *SREBP-1c*, *ACC*, and *FAS*, whereas it was positively (*p* < 0.05) correlated with the expression of *CPT1* and *PPARα*. The hepatic SOD level was negatively (*p* < 0.05) correlated with the expression of *ACC* and *FAS*, whereas it was positively (*p* < 0.05) correlated with the expression of *CPT1*. The hepatic *Nrf2* expression level was negatively (*p* < 0.05) correlated with the expression of *SREBP-1c*, whereas it was positively correlated with the expression of *CPT1*. The hepatic *HO-1* expression level was negatively correlated with the expression of *SREBP-1c*, whereas it was positively correlated (*p* < 0.05) with the expression of *PPARα*. The hepatic *GPX1* expression level was negatively (*p* < 0.05) correlated with the expression of *SREBP-1c*, *ACC*, and *FAS*, whereas it was positively (*p* < 0.05) correlated with the expression of *CPT1*. The hepatic *CAT* expression level was negatively (*p* < 0.05) correlated with the expression of *SREBP-1c* and *FAS*, whereas it was positively correlated (*p* < 0.05) with the expression of *PPARα* and *CPT1*.

The hepatic *IL-1β* expression level was positively (*p* < 0.05) correlated with the expression of *FAS* and *ELOV6*, whereas it was negatively correlated (*p* < 0.05) with the expression of *LPL*, *MTTP*, *PPARα*, and *CPT1*. The hepatic *IL-6* expression level was positively correlated (*p* < 0.05) with the expression of *SREBP-1c* and *FAS*, whereas it was negatively correlated (*p* < 0.05) with the expression of *LPL*, *MTTP,* and *PPARα*. The hepatic *IL-10* expression level was negatively (*p* < 0.05) correlated with the expression of *SREBP-1c*, whereas it was positively (*p* < 0.05) correlated with the expression of *CPT1*.

## 4. Discussion

Numerous studies have indicated that heat stress induces hormone and metabolic disorders, and poor growth performance in broilers [6,35,36]. Decreasing feed intake was found to be the primary physiological response to heat stress, as a defense mechanism to reduce heat production [37]. Heat stress negatively affected growth performance and was associated with poor feed intake, nutrient absorption and utilization, and intestinal health status [8,38,39,40]. Our former studies indicated that heat stress resulted in poor ADG, ADFI, and FCR in broilers [4,6]. Consistently, in this study, heat stress induced poor final BW, ADG, ADFI, and FCR. Meanwhile, we also found that COS supplementation could alleviate heat-stress-induced poor growth performance by increasing final BW, ADG, ADFI, and feed efficiency. As a functional oligosaccharide, previous studies illustrated that COS could promote the growth performance of broilers both under thermoneutral and heat-stressed condition by improving the ADFI, intestinal function, and health status, such as through better nutrient utilization, intestinal morphology, microbiology, barrier function, oxidative status, and immunity [6,29,30,41,42,43,44].

As the key metabolic and detoxification organ, the liver is sensitive to heat stress [45,46]. Former studies have indicated that heat stress induces different degrees of liver damage by causing oxidative stress and inflammatory response [16]. Generally, liver damage is accompanied by increasing serum ALT and AST levels [47]. Both chronic and acute heat stress increased serum ALT and AST levels [4,48]. Consistently, in this study, heat stress increased serum ALT and AST levels, suggesting the presence of liver damage in heat-stressed broilers. COS supplementation could alleviate heat-stress-induced liver damage by decreasing serum ALT and AST levels. Similar results were also reported by Chang et al. [6], who indicated that COS inhibited heat-stress-induced higher serum ALT and AST levels in yellow-feather broilers.

MDA is the end product of lipid peroxidation, and is applied as a biomarker to evaluate lipid peroxidation [49]. Anti-oxidant enzymes, such as GSH-Px, SOD, and CAT, are the first line of defense against ROS-induced oxidative damage [49]. It is undeniable that heat stress leads to oxidative damage in various tissues, including the liver of broilers [10,16,40,46]. Similarly, in this study, HS increased the liver MDA content, and decreased the liver GSH-Px and SOD activity in broilers. COS exerted an excellent anti-oxidant capacity by scavenging free radicals and terminating free radical reactions [50]. Lochi et al. [44] reported that chitosan could reduce the serum MDA level, and increase serum GSH-Px and SOD activity in heat-stressed broilers. Moreover, our previous studies demonstrated that COS supplementation could alleviate heat-stress-induced oxidative damage in the spleen, liver, breast muscle, and small intestine of yellow-feather broilers [30,31,51]. Consistently, in this study, COS supplementation decreased serum and liver MDA levels, and increased serum SOD and CAT activity, and liver GSH-Px activity, suggesting that COS could alleviate heat-stress-induced oxidative damage in broilers.

*Nrf2*, the key transcription factor in the antioxidant system, played an indispensable role in defending oxidative stress by regulating anti-oxidant enzyme-related gene expression [52]. In this study, heat stress down-regulated the expression of hepatic *Nrf2* and *GPX1* in broilers. These results suggest that heat stress inhibited the activation of the *Nrf2* signaling pathway, which, in turn, down-regulated the expression of *Nrf2* and *GPX1*, and decreased GSH-Px and SOD activity, thereby inducing oxidative damage in the liver. These results are supported by Tang et al. [10] and Ding et al. [16], who found that heat stress down-regulated the expression of hepatic *Nrf2*, *HO-1*, and *SOD*. Previous studies found that COS promoted *Nrf2* translocation and anti-oxidant-related gene expression in various cells and animal models [29,52,53]. Our former studies also indicated that COS supplementation increased the expression of *HO-1* in the breast muscle of broilers under acute heat stress [31]. In agreement with previous studies, in this study, COS supplementation up-regulated the expression of *Nrf2* and *CAT*, suggesting that COS alleviated heat-stress-induced oxidative stress by activating the *Nrf2* signaling pathway, and up-regulated the expression of *CAT*, thus enhancing anti-oxidant capacity in the liver. Additionally, the correlation analysis results demonstrated that the hepatic-lipogenesis-related gene expression (*SREBP-1c*, *ACC*, *FAS*) was negatively correlated with the anti-oxidant capacity (GSH-Px, SOD, *Nrf2*, *HO-1*, *GPX1*, *CAT*), whereas it was positively correlated with the MDA level. However, hepatic-lipolysis-related gene expression (*LPL*, *PPARα*, *MTTP*, *CPT1*) was positively correlated with anti-oxidant capacity (GSH-Px, SOD, *Nrf2*, *HO-1*, *GPX1*, *CAT*), whereas it was negatively correlated with MDA level. These results suggested that the positive effects of COS on lipid metabolism were related to its anti-oxidant activity.

In response to heat stress, T-helper 1 cells effectively activated the cell-mediated immune response, resulting in the excessive production of pro-inflammatory cytokines, including IL-1β, IL-6, TNF-α, and IFN-γ [8,29,54]. In this study, heat stress up-regulated the expression of pro-inflammatory cytokines *IL-1β* and *IL-6*, whereas it down-regulated the expression of the anti-inflammatory cytokine *IL-10* in the liver of broilers. These results were supported by Chen et al. [46], who reported that heat stress induced higher TNF-α and IL-1β levels in the liver, as well as increased the expression of *TNF-α* and *IL-1β*. Furthermore, COS increases the anti-inflammatory capacity of mice, as Wei et al. [55] indicated that COS supplementation decreased the TNF-α level, and increased the IL-10 level in the intestines of heat-stressed mice. Mohyuddin et al. [56] indicated that COS supplementation decreased serum TNF-α and IL-6 levels, and increased the IL-10 level of heat-stressed mice. Bai et al. [23] and Tao et al. [28] also indicated that COS supplementation decreased hepatic IL-1β and IL-6 levels, as well as the expression of hepatic *IL-1β* and *IL-6* in mice fed a high-fat diet. In this study, COS supplementation down-regulated the expression of *IL-1β* and *IL-6*, whereas it up-regulated the expression of *IL-10* in the liver, suggesting that COS could alleviate the heat-stress-induced inflammatory response in the liver of broilers. Under heat stress, excessive ROS production activated *NF-κB*, which increased the hepatic pro-inflammatory cytokine IL-6 level, and decreased the anti-inflammatory cytokine IL-10 level [17]. Thus, the beneficial effects on the inflammatory response might be related to the better anti-oxidant capacity with COS supplementation in heat-stressed broilers. Additionally, the correlation analysis results demonstrated that hepatic-lipogenesis-related gene expression (*SREBP-1c*, *ACC*, *FAS*, *ELOVA6*) was positively correlated with pro-inflammatory cytokine (*IL-1β*, *IL-6*) expression levels, whereas it was negatively correlated with anti-inflammatory cytokine *IL-10* expression level. However, hepatic-lipolysis-related gene expression (*LPL*, *PPARα*, *MTTP*, *CPT1*) was negatively correlated with the pro-inflammatory cytokines (*IL-1β*, *IL-6*) expression levels, whereas it was positively correlated with anti-inflammatory cytokine *IL-10* expression level. These results suggested that the positive effects of COS on lipid metabolism were associated with its anti-inflammatory capacity.

Broilers under heat stress usually suffered from an energy supply deficit due to their decreased feed intake and increased output for survival [19]. Numerous studies indicated that heat stress had detrimental effects on lipid utilization by reducing lipolytic capacity, enhancing TG transports from the liver to extrahepatic tissues and abdominal adiposity [4,5,19,57]. Consistently, in this study, heat stress resulted in higher serum TG level, and absolute and relative weight of abdominal adipose, suggesting that heat stress increased TG transports from the liver to extrahepatic tissues and abdominal adiposity. Furthermore, COS supplementation could alleviate the increase in the absolute and relative weight of abdominal adipose in heat-stressed broilers, suggesting that COS supplementation alleviated heat-stress-induced excessive fatty acid synthesis and deposition. Consistent with our results, Li et al. [58] and Wang et al. [59] also demonstrated that COS supplementation reduced the relative weight of abdominal adipose.

In poultry, adipose tissue mainly serves the purpose of fat storage, and the liver is the main site of lipogenesis. Overall, over 95% of fatty acids de novo synthesis is completed in the liver [5]. Hepatic lipid metabolism affects serum lipid metabolism and abdominal adiposity [13,45]. Moreover, the abdominal adiposity depends on hepatic TG synthesis and TG transports from the liver to extrahepatic tissues. In this study, heat stress induced higher serum TG level in broilers. Abdominal adiposity depends on hepatic de novo lipogenesis and TG absorption; the increased serum TG level explained the increased absolute and relative weight of abdominal adipose in heat-stressed broilers. Similar results were also reported by Li et al. [19]. COS exerted a lipid-lowering effect, and COS supplementation decreased the serum TG level in heat-stressed broilers, suggesting that COS decreased abdominal adiposity.

Fat deposition is mainly dependent on the dynamic equilibrium between lipid catabolism and biosynthesis, and it is a complex process associated with changes in lipogenesis, secretion, transportation, or lipolysis-related gene expression [60,61]. *SREBP-1c* is a key regulator of lipid synthesis in the liver, and is involved in the activation of fatty acid synthesis genes, including *ACC*, *FAS*, *SCD*, and *ELOVA6* [62]. The activation of *SREBP-1c* induced higher lipogenesis in the liver; meanwhile, liver-specific *SREBP-1c* knockout decreased hepatic and serum TG levels in mice [63,64]. Meanwhile, *PPARα* serves as ligand-activated nuclear receptors, which participates in lipid metabolism by regulating the expression of genes involved in lipolysis, transportation, and fatty acid oxidation, including *LPL*, *MTTP*, and CPT1 [15]. Previous studies have indicated that heat stress induces higher hepatic *SREBP-1c*, *ACC*, and *FAS* expression levels, as well as a lower hepatic *CPT1* expression level in broilers [4,5,19]. Consistently, in this study, heat stress increased the expression of hepatic *SREBP-1c*, *ACC*, and *FAS*, whereas decreased the expression of hepatic *PPARα*, *MTTP*, and *CPT1*. Based on these results, we speculated that heat stress led to enhanced abdominal adiposity due to the up-regulation of the lipogenesis-related gene (*SREBP-1c*, *ACC*, and *FAS*) and down-regulation of the lipolysis-related gene (*PPARα*, *MTTP*, and *CPT1*) in the liver of broilers. COS was found to be absorbed via the intestine and distributed to the blood and liver, exerting a lipid-lowering effects [65]. Tao et al. [28] reported that COS could ameliorate hepatic lipid accumulation by down-regulating the expression of *SREBP-1c* and *FAS*, and up-regulating the expression of *PPARα* and *CPT1* in mice fed a high-fat diet. Consistently, in this study, COS supplementation decreased hepatic *SREBP-1c*, *ACC*, and *FAS* expression, whereas it increased hepatic *LPL*, *PPARα*, *MTTP*, and *CPT1* expression in heat-stressed broilers. Similar to our results, Pan et al. (2018) and Li et al. (2022) reported that COS supplementation inhibited liver lipogenesis by down-regulating *SREBP-1c* and *FAS* in obese rats and in HepG2 cells, respectively [21,22]. Li et al. (2016) and Wang et al. (2022) illustrated that COS supplementation up-regulated hepatic *LPL* and lipase expression in broilers [58,59]. Li et al. (2022) also indicated that COS up-regulated the expression of *PPARα* and *CPT1* in HepG2 cells. Their results suggest that COS inhibits lipogenesis and accelerates fatty acid oxidation by regulating the hepatic *SREBP-1c* and *PPARα* pathway in heat-stressed broilers.

## 5. Conclusions

In conclusion, our study indicates that COS supplementation brings beneficial effects to the growth performance and health status of the liver, and alleviates hepatic oxidative stress, inflammatory response, and lipid metabolism disorders in heat-stressed broilers. Particularly, COS supplementation alleviates hepatic lipid metabolism disorders by improving the anti-oxidant capacity, inhibiting the inflammatory response, suppressing the expression of the lipogenesis-related gene, and up-regulating the expression of the lipolysis-related gene. These results provide a theoretical basis for the use of COS in the treatment of heat-stress-induced hepatic lipid metabolism disorders in broilers.

## Figures and Tables

**Table 1:** Dietary chitosan oligosaccharide supplementation on growth performance and carcass traits in broilers under heat stress.

Item ^1^	CON	HS	HSC_600_	SEM ^2^	*p*-Value
Initial BW (aged 14-day), g	358.22	352.21	350.53	10.58	0.2836
Final BW (aged 42-day), g	2403.67 ^a^	1972.33 ^b^	2327.00 ^a^	40.41	0.0004
ADG, g	73.05 ^a^	57.86 ^b^	70.59 ^a^	1.50	0.0002
ADFI, g	126.25 ^a^	111.96 ^b^	121.67 ^a^	2.46	0.0004
FCR	1.73 ^a^	1.94 ^b^	1.73 ^a^	0.05	0.0083
Abdominal adipose weight, g	32.60 ^a^	43.66 ^b^	34.21 ^a^	0.87	0.0001
Relative abdominal adipose weight ^3^, %	1.16 ^a^	1.56 ^b^	1.16 ^a^	0.04	0.0001
Liver weight, g	67.56	69.52	66.80	4.72	0.8058
Relative liver weight ^3^, %	2.39	2.50	2.26	0.19	0.5596

^1^ BW, body weight; ADG, average daily gain; ADFI, average daily feed intake; FCR, feed conversion ratio; CON, basal diet and raised in temperature-controlled room (24 ± 1 °C); HS and HSC_600_ were raised in high-temperature-controlled room (34 ± 1 °C, 8 h/day and 24 ± 1 °C, 16 h/day) and fed the basal diet with 0 or 600 mg/kg chitosan oligosaccharides; ^2^ SEM, standard error of mean; ^3^ Calculated as the percentage of body weight; ^a, b^ Within the same row, means with different superscripts were significantly different (*p* < 0.05).

**Table 2:** Correlations between hepatic-lipid-metabolism-related gene expression and indices related to oxidative stress and inflammatory response in the liver of broilers.

Items ^1^	*SREBP-1c*	*ACC*	*FAS*	*SCD*	*ELOVA6*	*LPL*	*MTTP*	*PPARα*	*CPT1*
MDA	0.75 **	0.49 *	0.69 **	0.21	0.17	−0.50 *	−0.48 *	−0.58 *	−0.61 **
GSH-Px	−0.52 *	−0.52 *	−0.65 **	−0.17	−0.21	0.37	0.47	0.61 **	0.64 **
SOD	−0.46	−0.49 *	−0.51 *	−0.14	−0.16	0.15	0.21	0.45	0.64 **
CAT	−0.14	0.12	−0.17	−0.23	−0.12	0.11	0.13	−0.06	0.37
*Nrf2*	−0.45 *	−0.43	−0.34	−0.32	−0.19	0.31	0.39	0.20	0.55 *
*HO-1*	−0.72 **	−0.25	−0.39	−0.04	0.03	0.06	0.29	0.60 *	0.39
*GPX1*	−0.67 **	−0.66 **	−0.72 **	−0.18	−0.02	0.38	0.42	0.43	0.81 **
*SOD*	−0.10	−0.13	0.02	−0.18	0.10	−0.11	0.36	0.34	−0.06
*CAT*	−0.51 *	−0.21	−0.66 **	−0.13	−0.17	0.38	0.42	0.67 **	0.50 *
*IL-1β*	0.41	0.19	0.63 **	−0.19	0.64 **	−0.56 *	−0.71 **	−0.55 *	−0.52 *
*IL-6*	0.67 **	0.34	0.73 **	0.04	0.21	−0.57 *	−0.59 **	−0.57 *	−0.42
*TNF-α*	0.13	0.28	−0.13	0.28	−0.13	−0.02	−0.16	−0.03	−0.09
*IL-10*	−0.65 **	−0.44	−0.39	−0.34	0.15	0.13	0.07	0.44	0.64 **

^1^ *SREBP-1c*, sterol regulatory element-binding protein 1c; *ACC*, acetyl-coenzyme carboxylase; *FAS*, fatty acid synthase; *SCD*, stearoyl-CoA desaturase; *ELOVA6*, elongation of very long chain fatty acids 6; *LPL*, lipoprotein lipase; *MTTP*, microsomal triglyceride transfer protein; *PPARα*, proliferator-activated regulator α; *CPT1*, carnitine palmitoyltransferase-1; *Nrf2*, nuclear factor erythroid related factor 2; *HO-1*, heme oxygenase-1; GSH-Px/*GPX1*, glutathione peroxidase; SOD/*SOD*, superoxide dismutase; CAT/*CAT*, catalase; MDA, malondialdehyde; *IL*, interleukin; *TNF-α*, tumor necrosis factor-alpha. * *p* < 0.05; ** *p* < 0.01.

## Data Availability

The data presented in this study are available upon request from the corresponding author.

## Supplementary Materials

The following supporting information can be downloaded at: https://www.mdpi.com/article/10.3390/antiox12081497/s1, Table S1: Ingredient composition and nutrient content of diets; Table S2: Primer sequences for real-time PCR.
