# Supplementary material for: Chitosan Oligosaccharides Alleviate Heat-Stress-Induced Lipid Metabolism Disorders by Suppressing the Oxidative Stress and Inflammatory Response in the Liver of Broilers
Source: Antioxidants (Basel). 2023 Jul 27;12(8):1497. doi: 10.3390/antiox12081497 (PMC10451627; doi:10.3390/antiox12081497)
Supplement: Supplementary file 1 [file antioxidants-12-01497-s001.zip › antioxidants-2495973-supplementary.pdf]

Table S1. Ingredient composition and nutrient content of diets

Item	Starter (day 1 to 14)	Grower (day 15 to 42)
Ingredients, %		
Corn	53.00	55.00
Soybean meal, 43% crude protein	33.00	34.82
Wheat bran	4.60	2.00
Fish meal	2.00	-
Soybean oil	3.00	5.00
Shell powder	1.71	0.51
CaHPO4	1.60	1.60
L- Lys, 78.4%	0.10	0.10
DL-Met, 88%	0.20	0.18
Salt	0.30	0.30
Vitamin premix[1]	0.20	0.20
Mineral premix[2]	0.20	0.20
Calculated composition		
Metabolic energy, MJ/kg	12.44	12.82
Crude protein, %	20.87	19.92
Ca, %	1.01	0.93
Available P, %	0.46	0.44
Lys, %	1.22	1.30
Met, %	0.53	0.45
Met + Cys, %	0.87	0.72

[1] Provided per kilogram of complete diet: 12,800 IU vitamin A, 1600 IU vitamin D_3, 60 IU vitamin E, 1.6mg vitamin K_3, 0.12mg biotin, 50mg choline, 1.2mg folic acid, 32mg nicotinic acid, 16mg pantothenic acid, 4.8mg riboflavin, 2.4mg thiamine (B_1), 3.2mg vitamin B_6, and 0.03mg vitamin B_{12}.

[2] Provided per kilogram of diet: Mg, 79mg as manganese oxide; Zn, 60mg as zinc oxide; Cu,100mg as copper sulfate; Fe, 120mg as iron sulfate; I, 0.96mg as potassium iodine; Co, 0.16mg as cobalt sulfate and Se ,0.24mg as sodium selenite.

Table S2. Primer sequences for real-time PCR[1]

Gene1	Accession NO.	Primer sequence (5′ to 3′)
β-actin	NM_205518.1	F: ATCCGGACCCTCCATTGTC<br>R: AGCCATGCCAATCTCGTCTT
SREBP-1c	XM_015294109	F: AGGCGGAGGTGATGGAGAT<br>R: TCGGAGTCACTGCTGCTGTT
FAS	NM_205155	F: TTTGGTGGTTCGAGGTGGTA<br>R: CAAAGGTTGTATTTCGGGAGC
ACC	J03541	F: GCTTCCCATTTGCCGTCCTA<br>R: GCCATTCTCACCACCTGATTACTG
SCD	NM_204890	F: GTTTCCACAACTACCACCATACATT<br>R: CCATCTCCAGTCCGCATTTT
ELOVA6	NM_001031539	F: GGTGGTCGGCACCTAATGAA<br>R: TCTGGTCACACACTGACTGC
PPARα	AF163809	F: TTTAACGGAGTTCCA ATCGC<br>R: AACCCTTACAACCTTCACAAGC
MTTP	NM_001109784	F: GCAGATGGACAGAGTTGGCT<br>R: ACACCAAAAGTGCAAGGTGC
LPL	NM_205282	F: CCGATCCCGAAGCTGAGATG<br>R: ACATTCCTGTCACCGTCCAC
CPT1	AY675193	F: TAGAGGGCGTGGACCAATAA<br>R: TGGGATGCGGGAGGTATT
TNF-α	NM_204267.1	F: GCCTATGCCAACAAGTACACCT<br>R: GCCAAGTCAACGCTCCTG
IL-1β	NM_204524.1	F: CGCCGCTACCAGAGGGACTT<br>R: CCGGACCCAGTTGACCCCAT
IL-6	NM_204628.1	F: GATCCGGCAGATGGTGATAA<br>R: AGGATGAGGTGCATGGTGAT
IL-10	NM_001004414.2	F: GCTCTCCTTCCACCGAAACC<br>R: GGAGCAAAGCCATCAAGCAG
Nrf2	NM_205117.1	F: ATCACGAGCCCTGAAACCAA<br>R: GGCTGCAAAATGCTGGAAAA
HO-1	XM_205344.1	F: ACTTCTATGGCAGCAACT<br>R: AATAGCGGG GTAGGC
SOD	NM_205064.1	F: TTGTCTGATGGAGATCATGGCTTC<br>R: TGCTTGCCTTCAGGATTAAAGTGG
GPX1	NM_000581.4	F: GATGAGATCCTGAGAGTGGTGGAC<br>R: TCATCAGGTAAGGTGGGCACAA
CAT	NM_001031215.2	F: TACGGTTCTCCACTGTTGCTG<br>R: TGGATGAAGGATGGAAACAAC

[1] *SREBP-1c*, sterol regulatory element-binding protein 1c; *ACC*, acetyl-coenzyme carboxylase; *FAS*, fatty acid synthase; *SCD*, stearoyl-CoA desaturase; *ELOVA6*, elongation of very long chain fatty acids 6; *PPARα*, proliferator-activated regulator *α*; *CPT1,* carnitine palmitoyltransferase-1; *LPL*, lipoprotein lipase; *MTTP*, microsomal triglyceride transfer protein; *TNF-α*, tumor necrosis factor-alpha; *IL*, interleukin; *Nrf2*, nuclear factor erythroid related factor 2; *HO-1*, heme oxygenase-1; *GPX1*, glutathione peroxidase; *SOD*, superoxide dismutase; *CAT*, catalase.
